# The PRMT5 inhibitor EPZ015666 is effective against HTLV-1-transformed T-cell lines *in vitro* and *in vivo*

**DOI:** 10.3389/fmicb.2023.1101544

**Published:** 2023-02-02

**Authors:** Kyle Ernzen, Corrine Melvin, Lianbo Yu, Cameron Phelps, Stefan Niewiesk, Patrick L. Green, Amanda R. Panfil

**Affiliations:** ^1^Department of Veterinary Biosciences, Center for Retrovirus Research, The Ohio State University, Columbus, OH, United States; ^2^Department of Biomedical Informatics, College of Public Health, The Ohio State University, Columbus, OH, United States; ^3^Comprehensive Cancer Center and Solove Research Institute, The Ohio State University, Columbus, OH, United States

**Keywords:** PRMT5, EPZ015666, HTLV-1, transformation, xenograft, humanized mice, T-cell

## Abstract

Human T-cell leukemia virus type 1 (HTLV-1) is the infectious cause of adult T-cell leukemia/lymphoma (ATL), an extremely aggressive and fatal malignancy of CD4^+^ T-cells. Due to the chemotherapy-resistance of ATL and the absence of long-term therapy regimens currently available for ATL patients, there is an urgent need to characterize novel therapeutic targets against this disease. Protein arginine methyltransferase 5 (PRMT5) is a type II PRMT enzyme that is directly involved in the pathogenesis of multiple different lymphomas through the transcriptional regulation of relevant oncogenes. Recently, our group identified that PRMT5 is overexpressed in HTLV-1-transformed T-cell lines, during the HTLV-1-mediated T-cell immortalization process, and in ATL patient samples. The objective of this study was to determine the importance of PRMT5 on HTLV-1 infected cell viability, T-cell transformation, and ultimately disease induction. Inhibition of PRMT5 enzymatic activity with a commercially available small molecule inhibitor (EPZ015666) resulted in selective *in vitro* toxicity of actively proliferating and transformed T-cells. EPZ015666-treatment resulted in a dose-dependent increase in apoptosis in HTLV-1-transformed and ATL-derived cell lines compared to uninfected Jurkat T-cells. Using a co-culture model of infection and immortalization, we found that EPZ015666 is capable of blocking HTLV-1-mediated T-cell immortalization *in vitro*, indicating that PRMT5 enzymatic activity is essential for the HTLV-1 T-cell transformation process. Administration of EPZ015666 in both NSG xenograft and HTLV-1-infected humanized immune system (HIS) mice significantly improved survival outcomes. The cumulative findings of this study demonstrate that the epigenetic regulator PRMT5 is critical for the survival, transformation, and pathogenesis of HTLV-1, illustrating the value of this cellular enzyme as a potential therapeutic target for the treatment of ATL.

## Introduction

Human T-cell leukemia virus type 1 (HTLV-1) is an oncogenic retrovirus that infects an estimated 5–10 million people worldwide (Gessain and Cassar, 2012). HTLV-1 is the sole causative agent of adult T-cell leukemia/lymphoma (ATL), a highly aggressive malignancy of CD4^+^ T-cells (Yoshida et al., 1982), HTLV-1-associated myelopathy/tropical spastic paraparesis (HAM/TSP), a chronic neurodegenerative disease of the spinal cord (Gessain et al., 1985; Osame et al., 1986), and a large number of other inflammatory diseases such as dermatitis, uveitis, and conjunctivitis (Martin et al., 2014). HTLV-1-associated diseases develop after a prolonged clinical latency period of up to several decades. While the projected lifetime risk of developing ATL is roughly 5% in HTLV-1-infected individuals, the risk rises to 25% in perinatal HTLV-1 carriers (Malik and Taylor, 2019). Given this high incidence of tumors, HTLV-1 is considered one of the most oncogenic human pathogens known to date (Tagaya and Gallo, 2017). Unlike other T-cell malignancies, ATL is chemotherapy-resistant and patient prognosis is poor with a median survival time of less than 1 year after diagnosis. In addition, the heterogeneity of ATL poses a challenge to the selection and implementation of effective treatments (Panfil et al., 2016). Thus, the development of novel therapeutics against HTLV-1 infection and associated malignancy is required.

HTLV-1 infects CD4^+^ T-cells and causes a persistent life-long infection of the host. Viral gene expression is driven from viral promoters within the long terminal repeats (LTRs) located at the 5′ and 3′ ends of the viral genome. Several studies have shown that tight regulation of HTLV-1 gene expression enables the virus to immortalize infected target cells, avoid immune detection, and establish persistent infection *in vivo*. Leukemogenesis is driven by clonal expansion of infected T-cells, rather than infectious cell-to-cell spread, and is the result of the genetic and epigenetic cellular changes that accumulate through this process (Ratner, 2020). A large body of research has shown that the viral genes *Tax* and *Hbz* play an important role in leukemogenesis (Giam and Semmes, 2016). Comparatively, little is known about the genetic and epigenetic cellular changes that drive HTLV-1-mediated leukemogenesis.

Protein arginine methyltransferase 5 (PRMT5) is a type II PRMT enzyme that regulates the transcription of key regulatory genes by symmetric di-methylation (S2Me) of arginine residues on histone proteins (Karkhanis et al., 2011). PRMT5 methylation of histone and non-histone proteins has also been shown to affect splicing, signal transduction, and the DNA damage response. Increased expression of PRMT5 is relevant to the pathogenesis of both hematologic and solid tumors (Pal et al., 2004, 2007; Wang et al., 2008; Majumder et al., 2010; Yan et al., 2010), and in most cases, this upregulation is associated with poor patient survival. Genetic alterations in PRMT5 genes are rare and thus control of PRMT5 in cancer has emerged as an attractive cancer therapeutic. Our group was the first to demonstrate that PRMT5 is upregulated in transformed T-cell lines, HTLV-1-transformed T-cells, and ATL patient-derived peripheral blood mononuclear cells (PBMCs; Panfil et al., 2015). In addition, we found that shRNA-mediated knockdown of PRMT5 protein in HTLV-1-infected lymphocytes caused a significant reduction in cellular proliferation. Use of a novel first-generation PRMT5 inhibitor (CMP5) likewise significantly reduced cellular proliferation and viability, suggesting that PRMT5 enzymatic activity is critical to HTLV-1-infected cell survival.

Here, we investigate the importance of PRMT5 on HTLV-1-infected cell survival, T-cell transformation, and disease pathogenesis of HTLV-1. Inhibition of PRMT5 enzymatic activity with a cell-potent and orally bioavailable small molecule inhibitor (EPZ015666) resulted in selective *in vitro* toxicity of transformed T-cells. EPZ015666 treatment resulted in a dose-dependent increase in apoptosis in HTLV-1-transformed and ATL-derived T-cell lines compared to uninfected Jurkat T-cells. Using a co-culture model of infection and immortalization, we found that EPZ015666 is capable of blocking HTLV-1-mediated T-cell immortalization *in vitro*, indicating that PRMT5 enzymatic activity is essential during transformation of HTLV-1-infected T-cells. Administration of EPZ015666 following disease onset in both HTLV-1-infected humanized immune system (HIS) mice and HTLV-1 xenograft mice significantly improved survival outcomes and decreased tumor burden *in vivo,* respectively. The cumulative findings of this study demonstrate that the epigenetic regulator PRMT5 is critical for the survival, transformation, and pathogenesis of HTLV-1, illustrating the value of this cellular enzyme as a potential therapeutic target for the treatment of ATL.

## Results

### PRMT5 inhibition is selectively toxic in actively proliferating T-cell lines

Our group previously demonstrated that inhibition of PRMT5 enzymatic activity with a PRMT5 small molecule inhibitor called CMP5 or through shRNA-mediated PRMT5 protein knockdown resulted in significantly decreased cellular proliferation and viability in HTLV-1-transformed T-cell lines (Panfil et al., 2015). CMP5 is a novel, first-generation selective PRMT5 inhibitor designed to selectively block the symmetric di-methylation of H4R3 (S2Me-H4R3) by PRMT5 (Alinari et al., 2015). CMP5 was identified through virtual docking of candidate small molecules with low binding energy in SAM cofactor and arginine-binding pockets of PRMT5. In our studies, we found that higher concentrations of CMP5 (25–50 μM) were required to achieve a significant decrease in the viability and proliferation of HTLV-1-transformed T-cells. To both improve and expand upon our previous studies, we assessed the capacity of a next generation orally bioavailable PRMT5 inhibitor, called EPZ015666, to selectively induce cellular toxicity in HTLV-1-transformed T-cell lines *in vitro*. EPZ01566 is a potent peptide-competitive and SAM-cooperative inhibitor that displays more than 10,000-fold specificity for PRMT5 over other protein arginine methyltransferases (Chan-Penebre et al., 2015). Treatment of mantle cell lymphoma (MCL) cell lines with EPZ015666 led to cell death with biochemical half-maximal enzyme-inhibition concentrations (IC_50_) in the nanomolar range (Chan-Penebre et al., 2015). Of note, this same study found that EPZ015666 had dose-dependent antitumor activity in multiple MCL xenograft mouse models.

**Figure 1:**
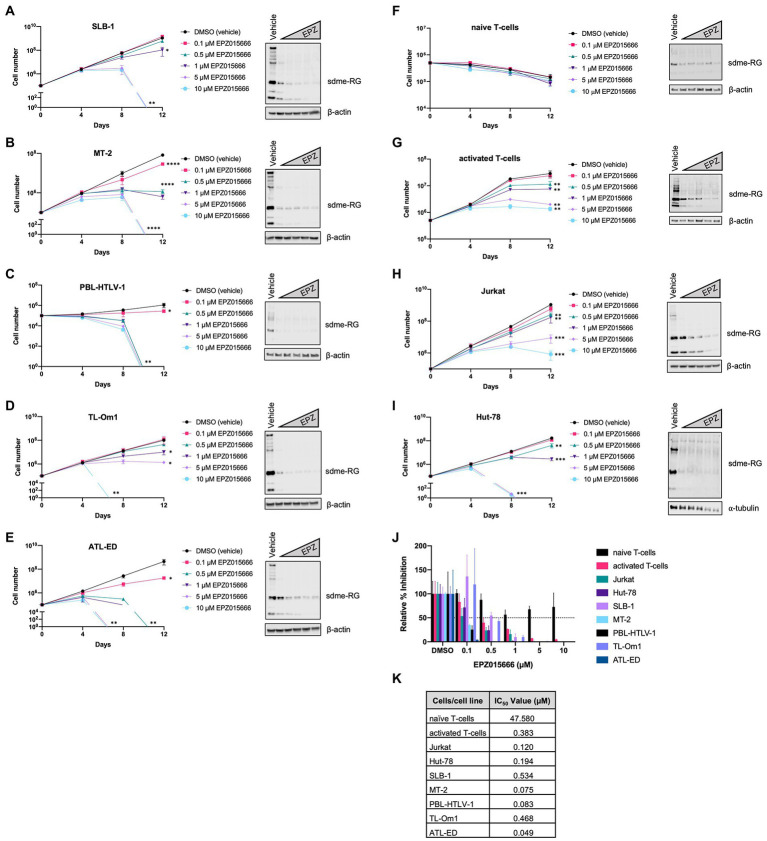
EPZ015666 is toxic to transformed T-cells. **(A)** SLB-1, **(B)** MT-2, **(C)** PBL-HTLV-1, **(D)** TL-Om1, **(E)** ATL-ED, **(F)** primary naïve CD4^+^ T-cells, **(G)** activated CD4^+^ T-cells, **(H)** Jurkat, and **(I)** Hut-78 cell lines were treated with titrating doses of EPZ015666 (0.1–10 μM) for 12 days (left panels). Viable cells were counted using Trypan blue dye exclusion. Each data point represents the mean for three replicates. Ordinary one-way ANOVA with Dunnett’s multiple comparison was used to determine significant differences between vehicle and EPZ015666-treated cells; *p* < 0.05 (*), *p* < 0.01 (**), *p* < 0.005 (***), *p* < 0.0001 (****). A portion of the cells were collected 4 days after EPZ015666 treatment and subjected to immunoblot analysis to compare the levels of Symmetric Di-Methyl Arginine Motif (sdme-RG) expression (right panels). β-actin and α-tubulin were used as loading controls. **(J)** Relative percent inhibition was calculated after 12 days of EPZ015666 treatment. **(K)** EPZ015666 IC_50_ values were calculated for each cell line/sample.

In order to evaluate the selective toxicity of EPZ015666 in HTLV-1-infected T-cells, a panel of HTLV-1-transformed, ATL-derived, and newly immortalized T-cell lines were treated with titrating amounts of EPZ015666 (0.1–10 μM) for 12 days. The antiproliferative effects of the inhibitor developed over several days, therefore a long-term proliferation assay was required. HTLV-1-transformed T-cell lines included SLB-1 and MT-2 (Figures 1A,B), PBL-HTLV-1 represents an infected and immortalized primary T-cell line (Figure 1C), and TL-Om1 and ATL-ED are patient ATL-derived T-cell lines (Figures 1D,E). EPZ015666 treatment of each HTLV-1-infected cell line led to a dose- and time-dependent decrease in cell viability. The effects of EPZ015666 treatment on total cellular symmetric arginine methylation (sdme-RG) in each cell line were examined by immunoblot after 4 days of treatment (Figures 1A–E, right panel). EPZ015666 treatment resulted in a concentration-dependent decrease in the intensity of multiple bands, indicating the effectiveness of the PRMT5 inhibitor. Cellular toxicity of EPZ015666 was next evaluated in normal resting CD4^+^ T-cells (Figure 1F), activated primary CD4^+^ T-cells (Figure 1G), and the HTLV-1-negative transformed T-cell lines, Jurkat (Figure 1H), and Hut-78 (Figure 1I). CD4^+^ T-cells represent the primary and preferential target of HTLV-1 infection (Richardson et al., 1990). EPZ015666 treatment of activated CD4^+^ T-cells, Jurkat, and Hut-78 cells led to a dose- and time-dependent decrease in cell viability. Interestingly, the inhibitor did not significantly affect the viability of normal resting CD4^+^ T-cells. The EPZ015666 IC_50_ concentrations for resting CD4^+^ T-cells were approximately 100-1,000-fold higher than that of HTLV-1-infected T-cell lines (Figure 1K). Collectively, these data demonstrate that PRMT5 inhibition with EPZ015666 is selectively toxic to activated, rapidly proliferating CD4^+^ T-cells (including HTLV-1-infected T-cell lines), while demonstrating limited toxicity to normal resting T-cells even after prolonged incubation (Figure 1J).

### Loss of PRMT5 enzymatic activity selectively induces apoptosis in HTLV-1-transformed cell lines

**Figure 2:**
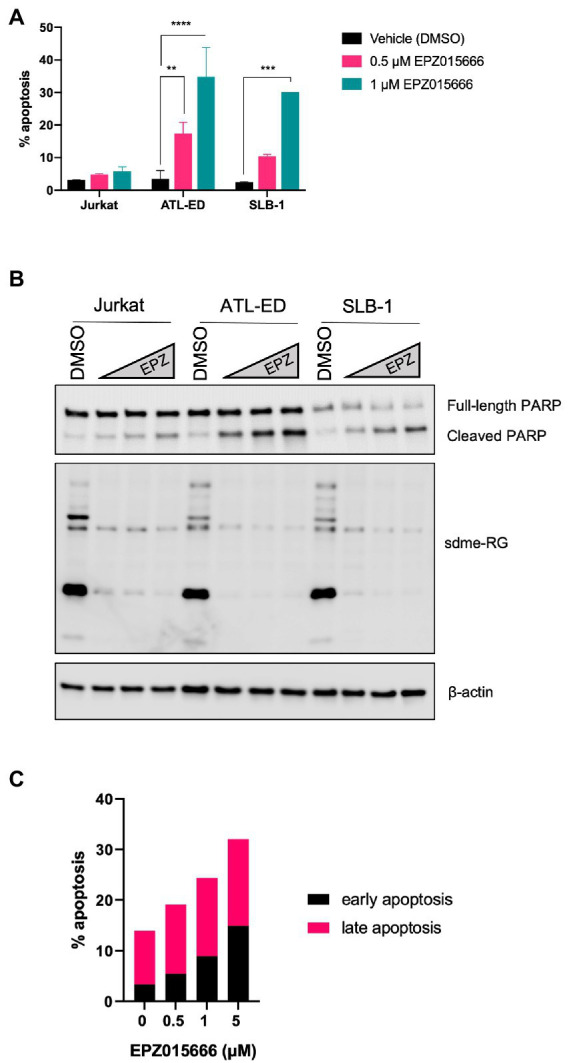
EPZ015666 treatment selectively increases apoptosis in HTLV-1-transformed cell lines. **(A)** Jurkat, SLB-1, and ATL-ED cells were treated with the indicated concentrations of EPZ015666. The level of total apoptosis was measured after 12 days of EPZ015666 treatment using a FITC Annexin V Apoptosis Detection Kit. Ordinary two-way ANOVA with Dunnett’s multiple comparison was used to determine significant differences between vehicle and EPZ015666-treated cells; *p* < 0.01 (**), *p* < 0.005 (***), *p* < 0.0001 (****). **(B)** Cell lysates were collected 4 days after EPZ015666 treatment and subjected to immunoblot analysis to compare the levels of sdme-RG and PARP/cleaved PARP expression. β-actin was used as a loading control. **(C)** ATL-ED cells were seeded in 6-well plates at a density of 2×10^5^ cells and treated with the indicated concentrations of EPZ015666. The amount of early vs. late apoptosis was measured in cells after 4 days of EPZ015666 treatment via flow cytometry analysis.

To further characterize the mechanism of EPZ015666-mediated cellular toxicity in HTLV-1-transformed T-cell lines, we examined the level of apoptosis in Jurkat (HTLV-1-negative), SLB-1 (HTLV-1-transformed), and ATL-ED (ATL patient-derived) cell lines. Cells were treated with titrating doses of EPZ015666 for 12-days. The level of apoptosis was examined in each cell population using flow cytometry and Annexin V staining for early apoptosis and Annexin V/propidium iodide (PI) staining for late apoptosis (Figure 2A). Total cellular symmetric arginine methylation (sdme-RG) in each cell line was examined by immunoblot after 4 days of treatment to confirm PRMT5 enzymatic inhibition (Figure 2A, right panel). We found no significant difference in the level of apoptosis in vehicle-treated compared to EPZ015666-treated Jurkat cells. In contrast, both SLB-1 and ATL-ED cell lines displayed a significant dose-dependent increase in apoptosis in EPZ015666-treated cells. To further confirm induction of apoptosis, we measured the expression of PARP in cells treated with EPZ015666 after 4 days (Figure 2B). PARP is a nuclear poly (ADP-ribose) polymerase that is involved in DNA repair in response to environmental stress. PARP helps cells to maintain their viability, such that cleavage of PARP facilitates cellular disassembly and thereby serves as a marker of cellular apoptosis. We found that EPZ015666 treatment of ATL-ED and SLB-1 cells led to a dose-dependent increase in PARP cleavage. We next compared the levels of early and late apoptosis in ATL-ED cells following a 4-day treatment with EPZ015666 (Figure 2C). We found that while EPZ015666 treatment led to an overall increase in apoptosis, it was the amount of early apoptosis that increased in an EPZ015666 dose-dependent manner.

### Inhibition of PRMT5 enzymatic activity blocks HTLV-1-mediated T-cell transformation

**Figure 3:**
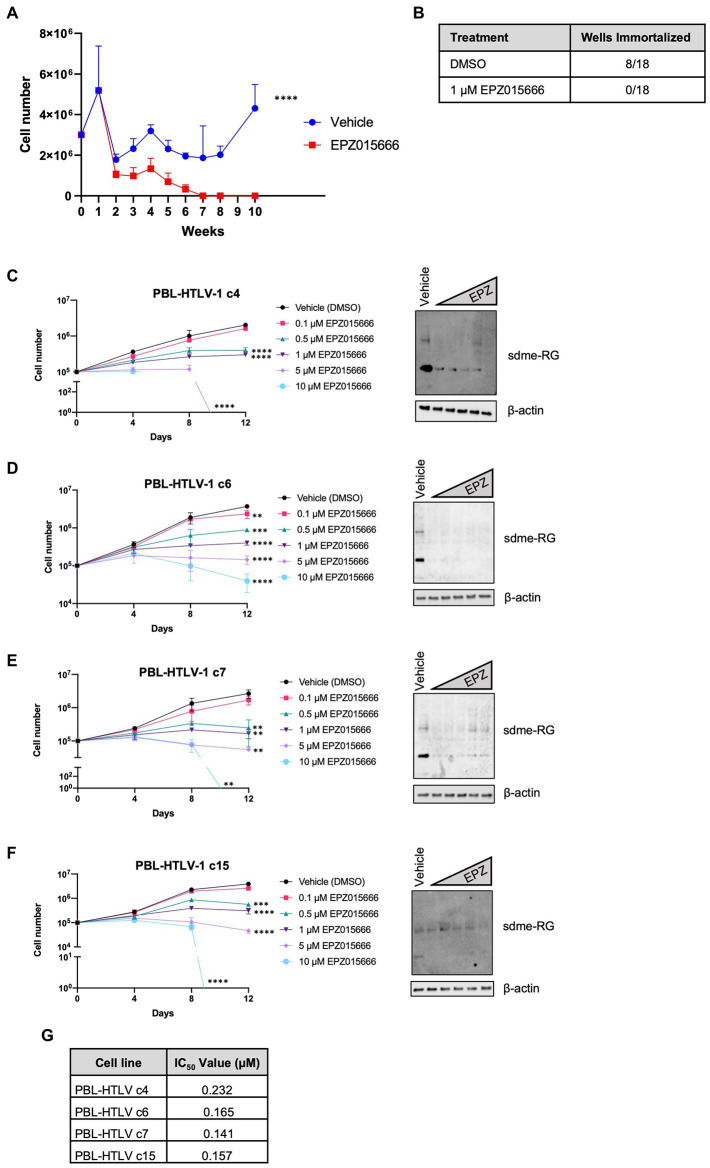
Loss of PRMT5 enzymatic activity blocks HTLV-1-mediated T-cell transformation. **(A)** Freshly isolated PBMCs (2 × 10^6^) were co-cultivated with 10^6^ lethally irradiated 729. ACH producer cells in 24-well plates. One week after co-culture, cells were treated with vehicle or 1 μM EPZ015666 at weekly intervals. Viable cell number was assessed weekly via Trypan blue dye exclusion from three random independent wells. Ordinary two-way ANOVA with Sidak’s multiple comparison was used to determine significant differences between vehicle and EPZ015666-treated cells; p < 0.0001 (****). **(B)** Cells which outgrew the co-culture were considered immortalized after 15 weeks. **(C–F)** Newly infected and immortalized PBL cell lines (i.e., less than 9 months old) were treated with titrating doses of EPZ015666 (0.1–10 μM) for 12 days (left panels). Viable cells were counted using Trypan blue dye exclusion. Each data point represents the mean for three replicates. Ordinary one-way ANOVA with Dunnett’s multiple comparison was used to determine significant differences between vehicle and EPZ015666-treated cells; *p* < 0.01 (**), *p* < 0.005 (***), *p* < 0.0001 (****). A portion of the cells were collected 4 days after EPZ015666 treatment and subjected to immunoblot analysis to compare the levels of Symmetric Di-Methyl Arginine Motif (sdme-RG) expression (right panels). β-actin was used as loading controls. **(G)** EPZ015666 IC_50_ values were calculated for each cell line.

Our group previously demonstrated that PRMT5 becomes dysregulated and overexpressed during the HTLV-1-driven T-cell transformation process (Panfil et al., 2015). Given these findings, we hypothesized that PRMT5 likely represents a key cellular factor that is required for the transformation of HTLV-1-infected T-cells. To test this hypothesis, we asked if enzymatic inhibition of PRMT5 would alter HTLV-1-driven T-cell transformation. Freshly isolated human PBMCs co-cultured with lethally irradiated HTLV-1 producer cells showed progressive growth over time, consistent with HTLV-1-mediated immortalization (Figure 3A). As a control, PBMCs co-cultured with lethally irradiated HTLV-1-negative cells were unable to sustain progressive growth (data not shown). However, PBMCs co-cultured with HTLV-1 producer cells in the presence of EPZ015666 were unable to sustain progressive cell growth over time. We further quantified the number of wells with cellular outgrowth in both the vehicle control and EPZ015666-treated co-culture groups at the end of the study (Figure 3B). Approximately 50% of our wells had sustained cellular outgrowth and became newly immortalized HTLV-1 T-cell lines, while 0% of the EPZ015666-treated wells were immortalized. This suggests that PRMT5 enzymatic activity is required for HTLV-1-mediated T-cell immortalization. Earlier results demonstrated that EPZ015666 was effective against an immortalized primary T-cell line called PBL-HTLV-1 (Figure 1C). To further characterize EPZ015666, we next examined the impact of PRMT5 inhibition on four separate newly immortalized (i.e., less than 9 months post-infection/immortalization) T-cell lines (Figures 3C–F). Each newly immortalized PBL cell line had dose-dependent sensitivity to EPZ015666, similar to results obtained with well-established HTLV-1-transformed T-cell lines (Figure 1). In addition, the IC50 values for each of the newly immortalized cell lines were similar to results obtained with HTLV-1-transformed T-cell lines (Figure 3G).

### EPZ015666 treatment increases survival and decreases tumor burden in HTLV-1 disease models *in vivo*

**Figure 4:**
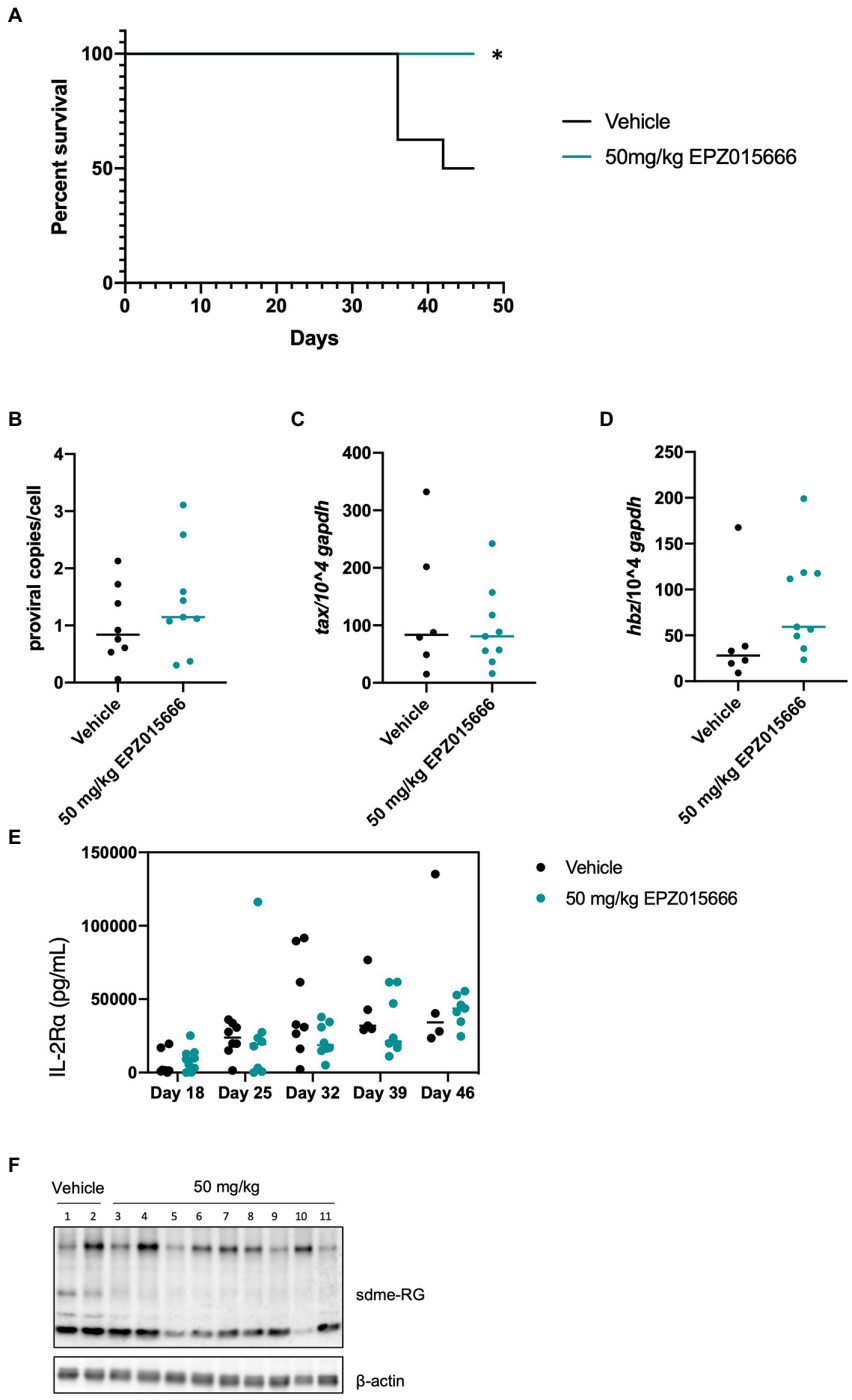
EPZ015666 increases survival in HIS mice. HIS mice were intraperitoneally inoculated with 1×10^6^ lethally irradiated HTLV-1 producer cells (729.ACH). At day 21 post-infection, mice were bidaily administered EPZ015666 (50 mg kg^−1^; *n* = 9) or vehicle (0.5% methylcellulose; n = 8) for the remainder of the study. Mice that had not succumbed to disease were sacrificed at day 46. **(A)** Percent survival data of each treatment group was represented as a Kaplan–Meier survival curve. Statistical significance was determined using the Log-rank Mantel–Cox test; p < 0.05 (*). **(B)** Proviral copies, **(C)** *tax* transcript, and **(D)** *hbz* transcript levels were measured from mouse spleen at the time of necropsy. **(E)** Human IL-2Rα levels were quantitated in mouse serum using an ELISA assay at 18-, 25-, 32-, 39-, and 46-days post-infection. **(F)** Splenic lysates were collected at time of necropsy and subjected to immunoblot analysis to compare the levels of sdme-RG expression. β-actin was used as a loading control.

HTLV-1 is unable to infect and replicate in murine cells (Delebecque et al., 2002). Therefore, the use of humanized immune system (HIS) mice is necessary to evaluate HTLV-1-mediated disease progression. HIS mice are created by intra-hepatic injection of CD34^+^ umbilical cord stem cells in NSG (NOD.Cg-*Prkdc^scid^ Il2rg^tm1Wjl^*/SzJ) mice. Subsequent analysis by flow cytometry and histology demonstrates normal circulation and population of lymphoid organs by human lymphocytes. Infection of these mice with HTLV-1 results in integration of the virus into the genome of human lymphocytes, followed by activation and proliferation of CD4^+^ T-cells. Subsequently, a CD4^+^ T-cell leukemia and lymphoma develops, which leads to eventual death of the mice (Maksimova et al., 2022). HTLV-1-infected HIS mice typically start to become moribund after 6–7 weeks. Using this accelerated HIS model of HTLV-1-mediated disease, we investigated if EPZ015666 treatment of infected HIS mice could impact disease development or survival outcomes. EPZ015666 has a favorable pharmacokinetic profile in mice with a reported oral bioavailability of 69% following oral administration of 10 mg kg^−1^ (Chan-Penebre et al., 2015). Twenty-one days after infection, HTLV-1-infected HIS mice were treated twice daily orally with of EPZ015666 (50 mg per kilogram of body weight [mg kg^−1^]). EPZ015666 was previously shown to be well tolerated in immune-deficient mice up to 200 mg kg^−1^ (Chan-Penebre et al., 2015) and we also detected minimal bodyweight loss in control mice (data not shown). EPZ015666-treated HTLV-1-infected HIS mice had significantly enhanced survival at study endpoint (46 days) compared to vehicle-treated mice (Figure 4A). At necropsy, splenic PBMCs were collected from each mouse, and proviral load (Figure 4B), viral sense transcript (*tax*; Figure 4C), and viral antisense transcript (*hbz*; Figure 4D) were measured using qPCR. Although proviral load and *hbz* transcript levels were slightly elevated in EPZ015666-treated mice, we found no significant differences in either proviral load or viral gene expression in vehicle compared to EPZ015666-treated mice. Serum was collected from each mouse throughout the course of study to measure human IL-2Rα levels. IL-2Rα is secreted by actively proliferating HTLV-1-infected cells and therefore, can be used as a biomarker for cellular proliferation *in vivo*. Using an IL-2Rα ELISA, we found no significant difference in the level of HTLV-1-infected cellular proliferation in vehicle vs. EPZ015666-treated mice (Figure 4E). To ensure *in vivo* target inhibition, we measured total cellular symmetric arginine methylation (sdme-RG) in each mouse spleen by immunoblot (Figure 4F). Although EPZ015666 treatment in HTLV-1-infected HIS mice increased survival, there was no significant difference in the proviral load, viral gene expression, or infected cell proliferation.

**Figure 5:**
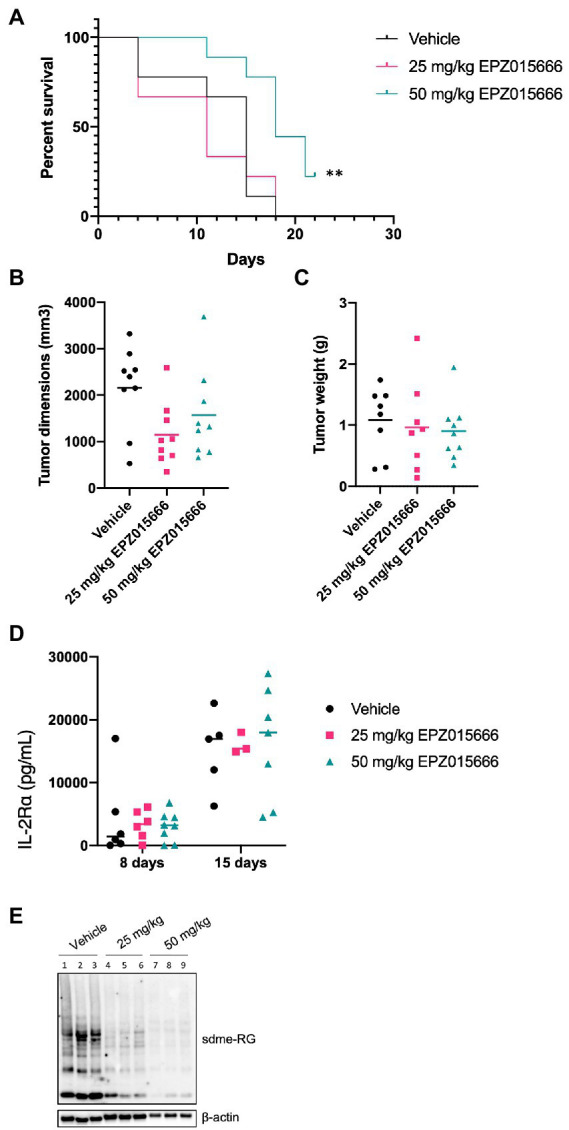
EPZ015666 increases survival in SLB-1 xenograft NSG mice. NSG mice were inoculated subcutaneously with 1×10^7^ SLB-1 cells. All the mice developed measurable tumors by day 8. Bidaily oral administration of EPZ015666 (25 mg kg^−1^, 50 mg kg^−1^; n = 9 per group) or vehicle (0.5% methylcellulose; *n* = 9) was subsequently initiated at day 9. Mice that had not succumbed to tumor burden were sacrificed at day 24. **(A)** Percent survival data of each treatment group was represented as a Kaplan–Meier survival curve. Statistical significance was determined using the Log-rank Mantel–Cox test; p < 0.01 (**). **(B)** Tumor dimensions and **(C)** tumor weight for each mouse was measured at time of necropsy. **(D)** Human IL-2Rα levels were quantitated in mouse serum using an ELISA assay at 8- and 15-days post-inoculation. **(E)** Tumor lysates were collected at time of necropsy and subjected to immunoblot analysis to compare the levels of sdme-RG expression. β-actin was used as a loading control.

**Figure 6:**
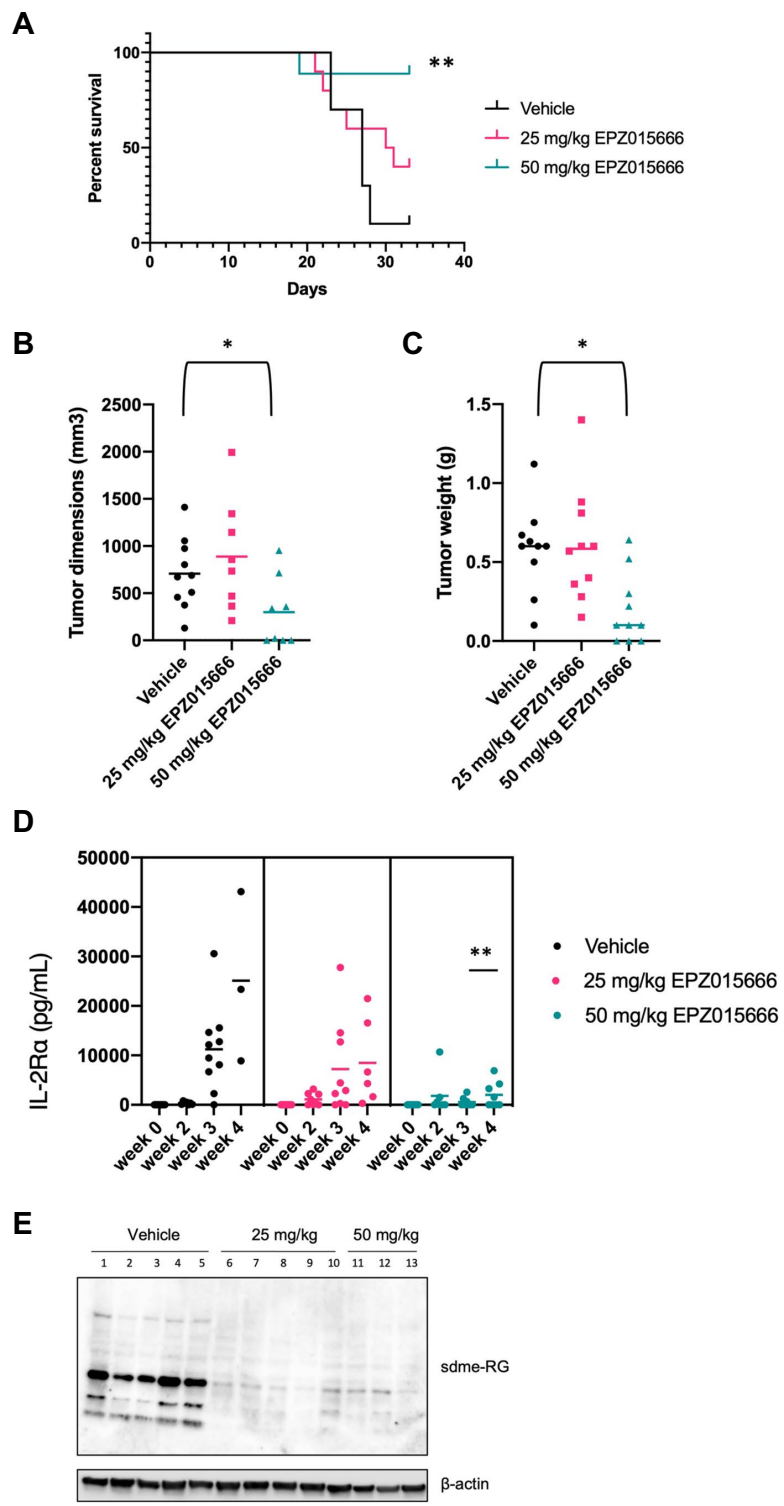
Inhibition of PRMT5 decreases tumor cell growth and increases survival in ATL-ED xenograft NSG mice. NSG mice were inoculated subcutaneously with 1×10^6^ ATL-ED cells. All the mice developed measurable tumors by day 11. Bidaily oral administration of EPZ015666 (25 mg kg^−1^, 50 mg kg^−1^; *n* = 9 per group) or vehicle (0.5% methylcellulose; *n* = 9) was subsequently initiated at day 12. Mice that had not succumbed to tumor burden were sacrificed at day 33. **(A)** Percent survival data of each treatment group was represented as a Kaplan–Meier survival curve. Statistical significance was determined using the Log-rank Mantel–Cox test; *p* < 0.01 (**). **(B)** Tumor dimensions and **(C)** tumor weight for each mouse was measured at time of necropsy. Welch’s unpaired t test was used to determine significant differences between vehicle and EPZ015666-treated cells; p < 0.05 (*). **(D)** Human IL-2Rα levels were quantitated in mouse serum using an ELISA assay at 0-, 2-, 3-, and 4-weeks post-inoculation. An unpaired *t* test was used to determine significant differences between vehicle and EPZ015666-treated cells; *p* < 0.01 (**). **(E)** Tumor lysates were collected at time of necropsy and subjected to immunoblot analysis to compare the levels of sdme-RG expression. β-actin was used as a loading control.

NSG mice characteristically lack mature T, B, and natural killer (NK) cells, resulting in impaired cytokine signaling and several deficiencies in their adaptive and innate immune response. This particular immune phenotype makes these mice an excellent model for the engraftment of human cells (Panfil et al., 2013). In an effort to further characterize PRMT5 as a viable therapeutic target for ATL patients, we next evaluated the efficacy of EPZ015666 treatment in NSG mice bearing subcutaneous SLB-1 (Figure 5) and ATL-ED (Figure 6) xenografts. NSG mice inoculated subcutaneously with HTLV-1-infected cell lines such as SLB-1 and ATL-ED will develop solid tumors (Dewan et al., 2003; Ohsugi et al., 2005; Arnold et al., 2008). SLB-1 and ATL-ED xenograft mice were treated with bidaily oral dosing of 25 or 50 mg kg^−1^ EPZ015666. Importantly, PRMT5 inhibitor was administered after the formation of small measurable tumors. EPZ015666-treated HTLV-1 xenograft mice had significantly enhanced survival compared to vehicle-treated mice (Figures 5A, 6A). 3-D tumor dimensions and tumor weight were measured at time of necropsy in each mouse. As expected, we found that EPZ015666-treated ATL-ED xenograft mice had decreased overall tumor burden (Figures 6B,C). We did not record a measurable difference in the overall tumor burden in SLB-1 xenograft mice at time of necropsy (Figures 5B,C). Serum was collected from each mouse throughout the course of study to measure human IL-2Rα levels. Using an IL-2Rα ELISA, we found no significant difference in the level of cellular proliferation in vehicle or EPZ015666-treated SLB-1 xenograft mice (Figure 5D). However, we observed a dose-dependent decrease in IL-2Rα levels in EPZ015666-treated ATL-ED xenograft mice (Figure 6D). To ensure *in vivo* target inhibition within the solid tumor, we measured total cellular symmetric arginine methylation (sdme-RG) in each mouse tumor by immunoblot (Figures 5E, 6E). Taken together, our results suggest that EPZ015666 can significantly decrease HTLV-1 solid tumor burden and increases overall survival in HTLV-1 xenograft mice.

## Discussion

HTLV-1 is an oncogenic retrovirus that preferentially infects and transforms CD4^+^ T-cells. Clonal expansion of infected T-cells is driven by viral gene expression and allows for the accumulation of genetic and epigenetic cellular changes, ultimately leading to the development of ATL. Currently, little is known regarding these genetic and epigenetic cellular changes that drive or regulate HTLV-1-mediated leukemogenesis.

PRMT5 is relevant to the pathogenesis of several hematologic and solid tumors (Pal et al., 2004, 2007; Wang et al., 2008; Majumder et al., 2010; Yan et al., 2010) and in most cases PRMT5 upregulation is associated with poor patient survival. Mutations in PRMT5 genes are rare and thus control of PRMT5 in cancer is a viable therapeutic strategy. In addition to upregulation in solid tumor and hematological malignancies (Poke et al., 2010), we previously found that PRMT5 is dysregulated in HTLV-1-transformed T-cells, lymphocytic leukemia T-cell lines, and ATL patient PBMCs. Furthermore, a decrease in PRMT5 protein levels or inhibition of PRMT5 enzymatic activity with a first-generation PRMT5 inhibitor (CMP5) in HTLV-1-infected T-cells resulted in decreased cellular proliferation and viability. The objective of our current study was to characterize the importance of PRMT5 on HTLV-1-infected cellular viability, T-cell transformation, and ATL disease pathogenesis using mouse models. Antagonization of PRMT5 enzymatic activity has demonstrated great promise in a multitude of pre-clinical cancer studies [reviewed in (Kim and Ronai, 2020). Therefore, we sought to provide insight into the potential efficacy of targeting PRMT5 for the treatment of HTLV-1 malignancies.

Our previous studies utilized a novel, first-in-class small molecule inhibitor of PRMT5 called CMP5 (Panfil et al., 2015). This inhibitor decreased survival in HTLV-1-infected T-cell lines and was effective at micromolar concentrations. Several other studies have described PRMT5 inhibitors with IC_50_ values in the micromolar range. A recent report described a cell-potent, orally bioavailable inhibitor of PRMT5 with nanomolar IC_50_ values for the treatment of MCL (Chan-Penebre et al., 2015). To further support PRMT5 as a viable therapeutic target, we evaluated if EPZ015666 had toxicity in a panel of HTLV-1-infected T-cell lines. For these studies, we utilized CD4^+^CD8^−^ HTLV-1-infected and HTLV-1-transformed T-cells, a subtype that typically comprises ~90% of HTLV-1-infected T-cells *in vivo* (Richardson et al., 1990). As a comparison, we also treated several HTLV-1-negative cells including Jurkat (CD4 T-cell line), Hut-78 (cutaneous T-cell line), and freshly isolated resting and activated CD4 T-cells from a healthy donor. After administering a comprehensive range of EPZ015666 across a 12-day period, we found that EPZ015666 had a dose-dependent cytotoxic effect on all HTLV-1-infected T-cell lines, HTLV-1-negative T-cell lines, and activated T-cells, but not resting T-cells (Figure 1). Importantly, EPZ015666 had IC_50_ values within the nanomolar range for all transformed T-cell lines. Taken together, these data suggest that EPZ015666 is cytotoxic against actively proliferating T-cells and could have therapeutic efficacy outside of HTLV-1-associated malignancies such as T-cell lymphoproliferative disorders. However, the cytotoxicity of EPZ015666 against activated T-cells must also be considered as a potential limitation of this drug.

The cytotoxic effect of EPZ015666 was further examined in SLB-1 (HTLV-1-transformed), ATL-ED (ATL patientderived), and Jurkat (HTLV-1-negative) T-cell lines by examining the level of apoptosis after PRMT5 inhibitor treatment. Other PRMT5 inhibitors have been shown to induce apoptosis in several different cancer cell lines (Kim and Ronai, 2020). Therefore, we suspected that EPZ015666 may induce apoptosis in transformed T-cell lines. Our results show that EPZ015666 preferentially induces apoptosis in HTLV-1-transformed T-cell lines, while by comparison, Jurkat cells have relatively little induction of apoptosis (Figure 2). This result suggests PRMT5 may regulate a cellular or viral factor critical to HTLV-1-infected cell survival, where loss of this factor induces apoptosis. Future experiments will be required to further dissect this mechanism.

Our group has previously reported that PRMT5 protein levels are dysregulated during HTLV-1-mediated T-cell transformation as soon as 1 week after viral infection (Panfil et al., 2015), suggesting that this cellular factor may be critical for the transformation of HTLV-1-infected T-cells. PRMT5 is also upregulated during EBV-mediated B-cell transformation and treatment with the PRMT5 inhibitor CMP5 prevented B-cell transformation (Alinari et al., 2015). To determine if PRMT5 is required for efficient T-cell transformation, we performed an *in vitro* co-culture infection and immortalization assay in the presence and absence of EPZ015666. The PRMT5 inhibitor was administered 1 week after viral infection to ensure no differences in viral infection. Our results show that EPZ015666 is able to block HTLV-1-mediated T-cell transformation (Figure 3) when treated early after infection. Importantly, we also show EPZ015666 is toxic against HTLV-1 newly immortalized CD4 T-cells. Future experiments which examine the timing of PRMT5 enzymatic inhibition may reveal at what point during the immortalization process PRMT5 activity is essential.

To our knowledge, there have been no studies investigating the role of PRMT5 in HTLV-1 pathogenesis. To assess if PRMT5 could potentially serve as an effective therapeutic target against HTLV-1 pathogenesis *in vivo*, we tested the efficacy of EPZ015666 in an ATL disease model in HIS mice and in HTLV-1 xenograft NSG mice. Immune-deficient NSG mice inoculated with HTLV-1-transformed cell lines, such as SLB-1 and ATL-ED, develop subcutaneous tumors. Relative to our vehicle-treated mice, low doses of EPZ015666 increased survival and reduced tumor burden in xenograft mice (Figures 5, 6). EPZ015666 treatment was especially effective in ATL-ED xenograft mice, a finding that correlates well with our *in vitro* selective toxicity studies (Figure 1).

Xenograft NSG mice characteristically lack T, B, and NK cells (Panfil et al., 2013), making this animal model substantially less suitable for studying new infection, persistence, and proliferation of infected T-cells *in vivo*. Conversely, HIS mice contain phenotypically normal T-cells which can be infected by HTLV-1. Treatment of HTLV-1 HIS mice with EPZ015666 allows us to examine the effect of PRMT5 inhibition during the course of disease development *in vivo*. Similar to the xenograft NSG mice, low-dose EPZ015666 treatment of HIS mice significantly extended their survival outcome (Figure 4). The proviral load and viral gene expression was examined in the splenic PBMCs of these mice after time of sacrifice. While we saw an increase in *hbz* transcript levels, this increase was not statistically significant between the groups of drug-treated mice. Limitations in the amount of blood that is able to be collected from mice at individual time points restrict our analysis of proviral load and gene expression to after euthanasia and splenic collection. Differences in sacrifice time (i.e., Day 36 vs. Day 44) may also account for the lack of significant difference in proviral load and viral gene expression. However, we were able to measure the level of secreted human IL-2Rα, a good indicator of T-cell proliferation, throughout the course of this study. Unfortunately, we did not observe a significant difference in IL-2Rα levels between vehicle and drug-treated mice over time. It is possible that EPZ015666-treated mice had a reduction in infected cells, but ongoing virus production and infection of new target cells in the absence of a functional immune response would counteract this effect. Another point to emphasize is that we elected to wait until 3 weeks after initial viral infection to ensure induction of T-cell proliferation and disease. It remains to be determined if pre-treatment of mice with EPZ015666 would have a more dramatic effect on survival and infected cell proliferation. Previous *in vivo* studies with EPZ015666 utilized a range of 25–200 mg kg^−1^ EPZ015666 for the treatment of MCL in xenograft mice (Chan-Penebre et al., 2015). We found a significant improvement in both HTLV-1 HIS mice and xenograft mice using lower doses (25 or 50 mg kg^−1^) of EPZ015666; however, our effects are somewhat modest suggesting that future studies should employ a wider and higher range of PRMT5 inhibitor concentrations. Altogether, our *in vivo* animal studies demonstrate that EPZ015666 reduces tumor burden and improves survival outcomes for both xenograft NSG mice and HIS mice inoculated with HTLV-1.

The primary objective of our study was to determine the importance of PRMT5 for HTLV-1-mediated T-cell transformation and disease pathogenesis *in vivo*, providing insight on the potential use of PRMT5 as a therapeutic target and PRMT5 inhibitors as therapeutics for the treatment of ATL. HTLV-1-mediated diseases arise after a prolonged clinical latency period, after the accumulation of several genetic and epigenetic cellular changes. The study of epigenetic factors during the course of HTLV-1 infection and disease remains a largely understudied area with vast potential for disease therapeutics. Future studies will examine the effect of PRMT5 on viral gene expression, the sole driver of early cellular proliferation in infected cells.

## Methods

### Cell lines

Human PBMCs (hPBMCs) were isolated from whole blood freshly collected from healthy donors using Ficoll-Paque PLUS (Cytiva, Marlborough, MA). Protocols for blood collection from human donors were approved by the Ohio State University Institutional Review Board. Naïve resting T-cells were enriched for CD4^+^ T-cells using a Human CD4 T Lymphocyte Enrichment Set (BD Biosciences, Franklin Lakes, NJ) according to the manufacturer’s instructions. CD4 T-cells were activated using the Dynabeads™ Human T-Activator CD3/CD28 kit (Thermo Fisher). Primary T-cells and early passage, HTLV-1-immortalized primary human T-cell lines (PBL-HTLV-1) were cultured in RPMI 1640 supplemented with 20% FBS, 100 U/ml penicillin, 100 μg/ml streptomycin, 2 mM L-glutamine, and 20 U/ml recombinant human interleukin-2 (hIL-2; Roche Diagnostics GmbH, Mannheim, Germany). Jurkat (HTLV-1-negative transformed T-cell line), Hut-78 (HTLV-1-negative transformed T-cell line), MT-2 (HTLV-1-transformed T-cell line), ATL-ED (ATL-derived T-cell line), and TL-Om1 (ATL-derived T-cell line) cells were maintained in RPMI 1640 supplemented with 10% FBS, 2 mM glutamine, 100 U/ml penicillin, and 100 μg/ml streptomycin. SLB-1 cells (HTLV-1-transformed T-cell line) and 729.ACH cells (HTLV-1 producer cell line) were maintained in Iscove’s medium supplemented with 10% FBS, 2 mM glutamine, 100 U/ml penicillin, and 100 μg/ml streptomycin. All cells were grown at 37°C in a humidified atmosphere of 5% CO_2_ and air.

### Immunoblotting

Cell lysates were harvested in NP-40 lysis buffer containing Complete Mini protease inhibitor cocktail (Roche Diagnostics). A small portion of each solid tumor (from NSG mice) or mouse spleen (from HIS mice) was collected in NP-40 lysis buffer containing Complete Mini protease inhibitor cocktail and homogenized using a BioMasher® II Micro Tissue Homogenizer (VWR). Protein quantification was carried out using the Pierce™ BCA Protein Assay Kit (Thermo Fisher) and a FilterMax F5 Multi-Mode Microplate Reader (San Jose, CA, United SA). Equal concentrations of protein were separated in Mini-PROTEAN® TGX™ Precast 4–20% Gels (Bio-Rad, Hercules, CA, USA) and transferred to nitrocellulose membranes. Membranes were blocked in PBS containing 5% milk and 0.1% Tween-20 and incubated with primary antibody overnight at 4°C. The following antibodies were used: anti-Symmetric Di-Methyl Arginine Motif [sdme-RG] (1:1000, Cell Signaling, Danvers, MA, USA), anti-PARP (1:1000, Cell Signaling), anti-β-actin (1:5000, Sigma, St. Louis, MO, USA), and anti-α-tubulin (1:250, Santa Cruz Biotechnology). Secondary antibodies used include IgG HRP anti-rabbit and anti-mouse (1:5000, Promega). Blots were developed using Pierce™ ECL Western chemiluminescence substrate (Thermo Scientific). Images were captured using the Amersham Imager 600 (GE Healthcare Life Sciences).

### Cell viability assays

Activated and naïve T-cells were seeded in 12-well plates at 5 × 10^5^ cells/mL and all other lymphoid cells were seeded in 12-well plates at 1 × 10^5^ cells/mL. EPZ015666 (MedChemExpress) or vehicle (DMSO) were added to duplicate wells at the indicated concentrations. Cells were incubated at 37°C. Cell viability was measured on days 4, 8, and 12 using Trypan blue dye exclusion. Fresh EPZ015666 or vehicle were added to the appropriate wells at the indicated concentrations every 4 days.

### Annexin V staining

Lymphoid cells were seeded in 6-well plates at 0.5 × 10^5^ cells/mL. EPZ015666 or vehicle (DMSO) were added to duplicate wells at the indicated concentrations on days 0, 4, and 8. Cells were incubated at 37°C for 12 days. Following incubation, cells were collected by slow centrifugation (5 min, 3,000 rpm) for apoptosis analysis *via* flow cytometry. Collected cells were stained using the FITC Annexin V Apoptosis Detection Kit I (BD Biosciences, Franklin Lakes, NJ, USA) according to the manufacturer’s instructions. Early apoptosis was defined was cells which stained positive for Annexin V (FITC) and negative for PI. Late apoptosis was defined as cells which stained positive for Annexin V (FITC) and PI.

### Co-culture immortalization assay

Long-term immortalization assays were performed as detailed previously (Green et al., 1995). Briefly, 2 × 10^6^ freshly isolated human PBMCs were co-cultivated at a 2:1 ratio with lethally irradiated 729.ACH cells (HTLV-1-producer cell line) in 24-well culture plates (media was supplemented with 20 U/ml rhIL-2). At 1-week post-infection (co-culture), cells were treated weekly with 1 μM EPZ015666 or vehicle (DMSO). Viable cells were enumerated weekly using Trypan blue dye exclusion.

### Humanized immune system (HIS) mice

Breeding pairs of NOD.Cg-*Prkdc^scid^ Il2rg^tm1Wjl^*/SzJ mice (NSG strain, specific pathogen free) were purchased from The Jackson Laboratory (Bar Harbor, ME). These mice lack mature T-cells, B-cells, or functional NK or dendritic cells, and are deficient in cytokine signaling. Animals were housed in individually ventilated microisolation cages with corncob bedding and provided with commercial pelleted rodent chow and reverse-osmosis–purified water without restriction. Cages containing autoclaved bedding were used for cage changes, which were performed in a ventilated biosafety cabinet. Mice were maintained in a room with constant temperature (20 ± 2°C) and relative humidity (50% ± 20%) under a 12:12-h light–dark cycle. The animal use protocol received prior approval by the Institutional Animal Care and Use Committee of The Ohio State University.

Shortly (24 to 48 h) after birth, pups were removed temporarily from the dam and treated with whole-body irradiation at 1 Gy (RS 2000, Rad Source, Suwanee, GA). Each mouse then was injected into the liver with 3 × 10^4^ to 1 × 10^5^ CD34^+^ HUSC (Lonza, Allendale, NJ) in 50 μl PBS (pH 7.4) by using a sterile 26-gauge hypodermic needle. After recovery, pups were returned to their dams, allowed to mature normally, weaned at 21 days, and then housed in groups (maximum, 5 mice per cage). At 10 weeks, after HUSC engraftment, mice were tested for the presence of human peripheral blood cells. Mice with at least 15% human CD45-positive lymphocytes were infected by intraperitoneal inoculation of 10^6^ lethally irradiated (100 Gy) 729.ACH producer cells; an aliquot of cells was maintained in culture to control for irradiation treatment. Mice began bidaily administration of vehicle (0.5% methylcellulose) or PRMT5 inhibitor (50 mg kg^−1^ EPZ015666) *via* oral gavage (n = 8–9 per treatment group) at day 21 post-infection. Mouse serum was collected at weekly time points by submandibular bleeds. Serum was used for quantification of human IL-2Rα levels using a human CD25/IL-2Rα Quantikine ELISA kit (R&D Systems, Inc., Minneapolis, MN). Animals were euthanized when they lost more than 20% of their body weight within 48 h.

### Xenograft NSG mice

The NOD.Cg-*Prkdc^scid^Il2rg^tm1Wjl^* /SzJ (NSG) xenograft mice were purchased from Charles River. NSG mice were subcutaneously inoculated with 1 × 10^7^ SLB-1 or 1 × 10^6^ ATL-ED cells. Mice were treated through bidaily administration of vehicle (0.5% methylcellulose) or PRMT5 inhibitor (25, 50 mg kg^−1^ EPZ015666) *via* oral gavage (n = 9 per treatment group) once palpable tumors were observed (8 days post-injection for SLB-1, 11 days post-injection for ATL-ED). Mouse serum was collected at weekly time points by submandibular bleeds. Serum was used for quantification of human IL-2Rα levels using a human CD25/IL-2Rα Quantikine ELISA kit (R&D Systems, Inc., Minneapolis, MN). At 24 days post-infection for SLB-1 and 33 days post-infection for ATL-ED, all remaining mice were euthanized. Tumors were extracted for assessment of weight (g) and dimensions (mm^3^). Animals were euthanized when they lost more than 20% of their body weight within 48 h.

### Quantitative PCR

Genomic DNA and RNA were isolated from HIS mouse spleens using the AllPrep DNA/RNA Mini Kit (QIAGEN, Hilden, Germany) according to manufacturer’s instructions. RNA samples were subjected to on-column DNase digestion, and 250 ng of RNA was used for cDNA synthesis, as described above. Approximately 1–2 μl cDNA was used per qPCR reaction with iQ™ SYBR® Green Supermix (Bio-Rad, Hercules, CA) and 300 nM of each sense and antisense primer (20 μl final reaction volume). Primer pairs to specifically detect viral mRNA species (tax, hbz) and gapdh were described previously (Li and Green, 2007). The reaction conditions were 95°C for 5 min followed by 40 cycles of 94°C for 30 s and 60°C for 45 s. Total copy numbers of each gene target in HIS mice were determined by log_10_ dilutions of plasmid DNA to generate a standard curve. Copy numbers were normalized to 1 × 10^4^ human GAPDH. Samples and standards were run in duplicate with no-RT and no-template controls included on each plate. To determine proviral load, 250 ng genomic DNA was used for qPCR with a primer set specific to HTLV-1 gag/pol. The 20 μl final reaction volume included iQ™ SYBR® Green Supermix and 300 nM each of 5′ primer (#20) and 3′ primer (#19). The reaction conditions were as follows: 95°C for 3 min followed by 40 cycles of 94°C for 30 s, 60°C for 45 s, and 72°C for 60 s. Total copy number was calculated using a standard curve generated by duplicate log_10_ dilutions of ACHneo plasmid DNA. Proviral copies per cell in HIS mice was calculated based on the approximation that 6 pg. human DNA is equivalent to 1 cell.

## Data availability statement

The original contributions presented in the study are included in the article/supplementary material, further inquiries can be directed to the corresponding author.

## Ethics statement

The studies involving human participants were reviewed and approved by the Ohio State University Institutional Review Board. The patients/participants provided their written informed consent to participate in this study. The animal study was reviewed and approved by the Ohio State University Institutional Animal Care and Use Committee.

## Author contributions

AP conceived and designed the study. KE and AP performed the experiments with support from CM, analyzed the data, and wrote the manuscript. CM and CP performed the experiments in mice with supervision from AP, PG, and SN. LY provided statistical support. AP, SN, and PG received funding to support the experiments. All authors critically reviewed and approved the manuscript.

## Funding

This work was supported by National Cancer Institute P01CA100730 to PG, NIH T32 GM141955 to KE, and The Ohio State Comprehensive Cancer Center.

## Conflict of interest

The authors declare that the research was conducted in the absence of any commercial or financial relationships that could be construed as a potential conflict of interest.

## Publisher’s note

All claims expressed in this article are solely those of the authors and do not necessarily represent those of their affiliated organizations, or those of the publisher, the editors and the reviewers. Any product that may be evaluated in this article, or claim that may be made by its manufacturer, is not guaranteed or endorsed by the publisher.
